# Establishing an open and robotic pancreatic surgery program in a level 1 trauma center community teaching hospital and comparing its outcomes to high-volume academic center outcomes: a retrospective review

**DOI:** 10.1186/s12893-022-01867-7

**Published:** 2022-12-06

**Authors:** Jane S. Han, C. Michael Dunham, Charles E. Renner, Steven A. Neubauer, F. Nikki McCarron, Thomas J. Chirichella

**Affiliations:** 1 Department of Surgery, Bon Secours Mercy Health St. Elizabeth Youngstown Hospital, 1044 Belmont Ave., Youngstown, OH 44504 USA; 2 Department of Surgery, University Hospitals Cleveland Medical Center, 11100 Euclid Ave., Cleveland, OH 44106 USA

**Keywords:** Hepato-pancreato-biliary surgery, Retrospective review, Robotic surgery, Robotic pancreatic surgery, Level 1 trauma center, Community teaching hospital, Academic center, Outcomes

## Abstract

**Background:**

The debate of whether to centralize hepato-pancreato-biliary surgery has been ongoing. The principal objective was to compare outcomes of a community pancreatic surgical program with those of high-volume academic centers.

**Methods:**

The current pancreatic surgical study occurred in an environment where (1) a certified abdominal transplant surgeon performed all surgeries; (2) complementary quality enhancement programs had been developed; (3) the hospital’s trauma center had been verified; and (4) the hospital’s surgical training had been accredited. Pancreatic surgical outcomes at high-volume academic centers were obtained through PubMed literature searches. Articles were selected if they described diverse surgical procedures. Two-tailed Fisher exact and mid-P tests were used to perform 2 × 2 contingency analyses.

**Results:**

The study patients consisted of 64 consecutive pancreatic surgical patients. The study patients had a similar pancreaticoduodenectomy proportion (59.4%) when compared to literature patients (66.8%; P = 0.227). The study patients also had a similar distal pancreatectomy proportion (25.0%) when compared to literature patients (31.9%; P = 0.276). The study patients had a significantly higher American Society of Anesthesiologists physical status ≥ 3 proportion (100%) than literature patients (28.1%; P < 0.001). The 90-day study mortality proportion (0%) was similar to the literature proportion (2.3%; P = 0.397). The study postoperative pancreatic fistula proportion was lower (3.2%), when compared to the literature proportion (18.4%; P < 0.001; risk ratio = 5.8). The study patients had a lower reoperation proportion (3.1%) than the literature proportion (8.7%; mid-P = 0.051; risk ratio = 2.8). The study patients had a lower surgical site infection proportion (3.1%) than those in the literature (21.1%; P < 0.001; risk ratio = 6.8). The study patients had equivalent delayed gastric emptying (15.6%) when compared to literature patients (10.6%; P = 0.216). The study patients had decreased Clavien–Dindo grades III–IV complications (10.9%) compared to the literature patients (21.8%; mid-P = 0.018). Lastly, the study patients had a similar readmission proportion (20.3%) compared to literature patients (18.4%; P = 0.732).

**Conclusion:**

Despite pancreatic surgical patients having greater preoperative medical comorbidities, the current community study outcomes were comparable to or better than high-volume academic center results.

**Supplementary Information:**

The online version contains supplementary material available at 10.1186/s12893-022-01867-7.

## Background

The debate of whether to centralize hepato-pancreato-biliary (HPB) surgery has been ongoing for the past two decades. A higher case volume leads to better surgical outcomes. HPB disease often requires multidisciplinary care teams and specialized resources with centralization, which has naturally gravitated toward large academic medical centers. However, access to care remains to be an issue, whether it be due to patients’ unwillingness or inability to travel. As a level 1 trauma center with a community teaching program located 70 miles away from two major cities home to large volume HPB programs, its primary care physicians witness various dilemmas patients face to decide where to ultimately receive their operative as well as pre- and postoperative HPB care.

In our experience, the intangible factors that affect patients’ overall care become especially important in diseases such as pancreatic cancer. Establishing a new pancreatic surgery program in the community setting poses a unique set of challenges, but these do not necessarily outweigh the benefits. Community programs that were already long established in pancreatic surgery have shown equivalent outcomes to those of academic programs. In addition, recent evidence has suggested that improved outcomes have more to do with the individual surgeon’s experience than with the hospital’s total case volume, if that surgeon is specifically trained in HPB surgery [1, 2].

To help maintain and improve patients’ access to high-quality HPB care, more data are needed to support skilled and motivated surgeons’ desires to practice in communities of need rather than being limited to academic centers. Because of the increasing demand for minimally invasive surgery, careful consideration should be given to incorporating laparoscopic and robotic HPB surgery into community programs too. The training background of our hospital’s HPB surgeon enabled robotics to be incorporated as soon as our HPB program was established. The current study aimed to compare the outcomes collected to those of high-volume academic centers. We hypothesized that equivalent outcomes could be achieved in as early as the first few years of a newly established pancreatic surgery program in the community setting.

## Methods

### Ethics statements

This study was approved by the hospital’s institutional review board (IRB) (Mercy Health Youngstown, LLC IRB (FWA00001840); Approval #21-012). The need for informed consent was waived by the Mercy Health Youngstown, LLC IRB because of the retrospective nature of the investigation. All the methods were carried out in accordance with relevant guidelines and regulations.

### Study design and population

To compare outcomes of a community pancreatic surgical program with those of high-volume academic centers, we retrospectively reviewed all patients who underwent pancreatic surgery at a single institution by one surgeon (TJC) during the first 3 years of employment at Bon Secours Mercy Health St. Elizabeth Youngstown Hospital (SEYH) from February 2017 through March 2020. TJC is an HPB surgeon who completed an American Society of Transplant Surgeons accredited Abdominal Transplant Surgery fellowship and was proctored by an expert in robotic HPB surgery. SEYH is a community teaching hospital and level 1 trauma center associated with the Northeast Ohio Medical University general surgery residency program.

Upon the senior author’s arrival, an HPB multidisciplinary conference was established, and staff met weekly to discuss patients. Prior to the start of this program, there was little to no HPB surgery performed at the hospital outside of trauma. Each surgery was assisted by a senior resident from the general surgery residency program. Most of the distal pancreatectomy with splenectomy (DPS) surgeries and all the transgastric pancreatic necrosectomy with cyst gastrostomy (PNCG) surgeries were performed robotically using the DaVinci Xi platform (Intuitive Surgical, Inc., Sunnyvale, CA, USA), with one open necrosectomy with Roux-en-Y drainage. All pancreaticoduodenectomy (PD) procedures were performed open and consisted of classic and pylorus-preserving techniques. Additionally, a standard postoperative pathway implemented for pancreatic patients was followed daily by the attending surgeon, surgical residents, and nursing, social work, and dietary staff. A formal enhanced recovery after surgery program that also incorporated preoperative nutritional strategies was implemented in 2018 [3]. Each postoperative patient was either admitted to the surgical intensive care unit or a designated intermediate level floor to ensure consistency from a nursing care standpoint.

### Data collection and literature search

Data were collected prospectively, and outcomes evaluated during the first 90 days of surgery (except readmission, defined as within 30 days) were as follows: the operative time, estimated blood loss (EBL), intraoperative transfusion requirement, microscopic residual tumor resection (R1) (all others were R0 resection, without residual tumor) [4], reoperation, biochemical pancreatic leak, grade B or C postoperative pancreatic fistula (POPF), bile leak, surgical site infection (SSI), delayed gastric emptying (DGE), Clavien–Dindo grades III–IV complication (CD III–IV) [5], initial hospital length of stay (LOS), readmission, and all-cause mortality. Pancreatic leak and POPF were defined based on the 2016 International Study Group definition and grading [6]. The preoperative American Society of Anesthesiologists physical status (ASA) was routinely documented by a certified anesthesiologist. The results were compared to those of high-volume centers found in a PubMed literature search.

The literature search was conducted in PubMed to identify articles that describe pancreatic surgical procedures (PD, DPS, or PNCG) that had been performed in a high-volume academic center. Procedural outcomes from pancreatic surgical academic or referral services were considered to be high-volume academic center studies (HVAS). A text search was performed using the terms “pancreas” or “pancreatic” and “surgery” or “surgical.” Additional search qualifications were publication years 2010 to 2020, English language, and adult. The abstract of each article was reviewed to determine if any of the following proportion data were included in the manuscript: preoperative ASA ≥ 3, 30-day or 90-day mortality, POPF, reoperation, SSI, DGE, CD III–IV, or readmission. If any of these proportions were found in the manuscript and the manuscript showed that HVAS criteria had been met, the authors considered these outcomes to be representative of outcomes of HVAS and compared them to the current community study (CCS) outcomes. The final HVAS selection criterion was to include studies that had performed diverse pancreatic surgical procedures (PD and DPS). To facilitate the identification of these studies, two searches were performed using PubMed text terms. The first search used “pancreatic surgery” and “outcomes” and included the filters (1) the year of publication 2015 to 2021, (2) a focus on adult patients, and (3) the manuscript was written in English. A total of 2151 citations were identified. The second search used “pancreatic surgery” and “high-volume” and included the filters (1) the year of publication 2015 to 2021, (2) a focus on adult patients, and (3) the manuscript was written in English. A total of 491 citations were identified. From these two searches, abstracts were reviewed and manuscripts were considered to be initially appropriate if any relevant outcome data was provided and diverse pancreatic surgical procedures had been performed. Subsequently, manuscripts were reviewed and studies were included, if the facility met HVAS criteria and surgical procedures had been performed during the years 2000 to 2021. References from other articles were used as a potential source of literary discovery. Relevant data included the PD and distal pancreatectomy (DP) proportions of the HVAS to be compared with the CCS proportions.

### Statistical analyses

Continuous data are presented as the mean ± standard deviation. The CCS results were entered into Excel 2010 (Microsoft Corp., Redmond, WA, USA) and imported into SAS System for Windows, release 9.2 (SAS Institute, Inc., Cary, NC, USA). For univariate analyses with a dichotomous dependent outcome, intergroup mean differences were analyzed using the independent t-test for continuous data. For dichotomous proportional data, represented as a 2 × 2 contingency table, a two-tailed Fisher exact test was employed to assess the risk ratio. Correlation analysis of two continuous variables was performed using the Pearson correlation coefficient. The study results for each of the HVAS diverse pancreatic surgical study outcome proportions (PD, DP, ASA ≥ 3, 90-day mortality, POPF, reoperation, SSI, DGE, CD III–IV, and readmission) were entered into separate outcome tables. For each study that provided relevant data, the total number of patients and the outcome patient number were entered into the table. Separate sums were created for the total number of patients from HVAS assessed for a specific outcome. Each of the HVAS outcome results consisted of the number of patients without and with the outcome for each study. A total HVAS proportion was computed for each outcome by combining the proportions for each relevant study. When the test for proportion heterogeneity had a P < 0.05, the total random effects proportion was used. With a P ≥ 0.05, the total fixed effects proportion was used. Total proportions were computed in MedCalc® Statistical Software, version 19.2.6 (MedCalc Software Ltd, Ostend, Belgium). Each HVAS outcome proportion was computed by multiplying the total proportion times the total number of patients from each study. Then, the relevant CCS and total HVAS proportions were entered into a 2 × 2 contingency table. Comparisons between the CCS and HVAS were performed in Epi Info™ 7.0.9.7, a statistical analysis program developed by the Centers for Disease Control and Prevention [7]. The program computes a two-tailed Fisher exact P-value and a one-tailed mid-P-value for 2 × 2 contingency analyses. The mid-P-value was used if the value reached significance, yet the two-tailed Fisher exact P-value did not. Multiple statisticians have found the mid-P-value to be robust, reliable, and preferable for relatively small cohort analyses [8, 9]. All P-values in the Results section are two-tailed, unless otherwise qualified. The P-value of significance was < 0.05.

## Results

### All pancreatic surgical patients

A total of 128 patients received HPB surgery at our institution from February 2017 to March 2020. Of these, 64 had pancreatic surgery and were included in the present study. Patients’ demographics are described in Table 1. Procedures consisted of 38 PDs, 16 DPSs, seven PNCGs, one pancreatic necrosectomy with Roux-en-Y cyst jejunostomy (PNRCJ), one duodenum-preserving pancreatic head resection, and one total pancreatectomy. Forty-six of the 64 cases were performed open, and the 18 others (11 of 16 DPSs, 7 of 7 PNRCJs) were performed robotically. There were four R1 resections (13% of the cancer cases among PD and DPS) with zero R2 resections. Intraoperative measures and hospital LOS will be discussed separately.

**Table 1:** Patient characteristics

Demographic	N	%
All patients	64	–
Male sex	30	47
Smoker	48	75
COPD	10	16
Cardiac disease	13	20
Diabetes	15	23
BMI (kg/m^2^)	*26.6 (16–47)	–
Patients with PD	38	–
Male sex	18	47
Smoker	31	82
COPD	7	18
Cardiac disease	8	21
Diabetes	5	13
BMI (kg/m^2^)	*25.0 (16–47)	–

*BMI* body mass index, *COPD* chronic obstructive pulmonary disease, *PD* pancreaticoduodenectomy

*Group average (and range)

The average ASA was 3.3 ± 0.5. There was no mortality during the first 30 and 90 days. The data represented nine biochemical pancreatic leaks (14%) with only two grade B postoperative POPFs (3%), zero grade C postoperative POPFs, and zero bile leaks. There were two reoperations (3%), two SSIs (3%), 10 patients with DGE (16%), seven with CD III–IV (11%), and 13 hospital readmissions (20%).

The literature search found seven relevant mixed pancreatic surgical citations from HVAS [10–16]. The CCS patients had a similar PD proportion when compared to the patients of HVAS (Table 2; Additional file 1) [10–12, 14–16]. The CCS patients had a similar DP proportion when compared to the patients of HVAS (Table 2; Additional file 1) [10–12, 14–16]. The CCS patients had a significantly higher ASA ≥ 3 proportion than the patients of HVAS (Table 2; Additional file 2) [11, 12, 15, 16]. The CCS patients had a similar 90-day mortality proportion when compared to the patients of HVAS (Table 2; Additional file 3) [10, 15]. The CCS patients had a lower POPF proportion than the patients of HVAS (Table 2; Additional file 4) [10–16]. The CCS patients had a lower reoperation proportion than the patients of HVAS (Table 2; Additional file 5) [11, 12, 15]. The CCS patients had a lower SSI proportion than the patients of HVAS (Table 2; Additional file 6) [10, 12, 15]. The CCS patients had a similar DGE proportion compared to the patients of HVAS (Table 2; Additional file 7) [10–13, 15, 16]. The CCS patients had a lower CD III–IV proportion than the patients of HVAS (Table 2; Additional file 8) [11, 12, 14]. The CCS patients had a similar readmission proportion compared to the patients of HVAS (Table 2; Additional file 9) [10, 12, 14]. Test results for HVAS proportion heterogeneity and total proportion values are included in Additional files 1, 2, 3,  4,  5,  6,  7,  8,  9.

**Table 2:** Comparisons of the total study cohort with patients of HVAS

	CCS	HVAS	P-value	Mid-P	RR
PD %	38/64 (59.4%)	1347/2017 (66.8%)	0.227		
DP %	16/64 (25.0%)	643/2017 (31.9%)	0.276		
ASA ≥ 3%	64/64 (100%)	441/1571 (28.1%)	< 0.001		3.6
90-day mortality %	0/64 (0%)	32/1403 (2.3%)	0.397		
POPF %	2/63 (3.2%)	489/2658 (18.4%)	< 0.001		5.8
Reoperation %	2/64 (3.1%)	130/1498 (8.7%)		0.051	2.8
SSI %	2/64 (3.1%)	302/1431 (21.1%)	< 0.001		6.8
DGE %	10/64 (15.6%)	253/2385 (10.6%)	0.216		
CD III–IV %	7/64 (10.9%)	118/541 (21.8%)	0.049	0.018	2.0
Readmission %	13/64 (20.3%)	87/474 (18.4%)	0.732		

*HVAS* high-volume academic center studies, *CCS* current community study, *RR* risk ratio; *PD* pancreaticoduodenectomy, *DP* distal pancreatectomy, *ASA* American Society of Anesthesiologists physical status, *POPF* grade B or C postoperative pancreatic fistula, *SSI* surgical site infection, *DGE* delayed gastric emptying, *CD III–IV* Clavien–Dindo grades III–IV complication

### Patients with pancreaticoduodenectomy

Patients with PD comprised 30% of all HPB cases from February 2017 to March 2020 (Table 3). On average, 13 PDs were performed per year, including portal vein reconstructions and hepatic artery reconstructions when needed. Benign indications for surgery included mucinous cystic neoplasm, branch-duct intraductal papillary mucinous neoplasm, and chronic pancreatitis. The surgical indication was malignancy in 22 patients (58%); four of them had R1 resections (18%), whereas the remainder were R0 resections. A longer operative time was associated with R1 resection than with R0 resection (P = 0.045).

**Table 3:** Outcomes of patients with pancreaticoduodenectomy

Outcome	N	% of 38
R1 Resection	4	*18
Reoperation	2	5
Chem Leak	4	11
POPF	2	5
Bile Leak	0	0
SSI	2	5
DGE	10	26
Grade A	3	8
Grade B	5	13
Grade C	2	5
CD III–IV	5	13
Readmission	8	21
Mortality	0	0
Average OR Time	444 ± 76 min	–
Average EBL	697 ± 546 mL	–
Average PRBC	1.0 ± 1.6 units	–
Average LOS	8.0 ± 4.2 days	–
Median LOS	6 days	

*CD III–IV* Clavien-Dindo grades III–IV complication, *Chem Leak* biochemical pancreatic leak, *DGE* delayed gastric emptying, *EBL* estimated blood loss, *LOS* initial hospital length of stay, *OR Time* operative time, *POPF* grade B or C postoperative pancreatic fistula, *PRBC* packed red blood cells transfused intraoperatively, *SSI* surgical site infection

*This rate only applies to the 22 pancreaticoduodenectomies performed for cancer

The average overall operative time was 444 ± 76 min (range, 212–623 min), average EBL was 697 ± 546 mL (range, 50–2500 mL), and average intraoperative transfusion requirement was 1.0 ± 1.6 packed red blood cell units (range, 0–6 units). These averages improved over time from the first half to the latter half in our patients: from 486 to 403 min (P < 0.001), from 892 to 501 mL (P = 0.025), and from 1.4 to 0.6 units (P = 0.139), respectively. Older age was associated with increased transfusions (P = 0.024), perhaps due to a natural decline in cardiac reserve with age. Cardiac disease had a positive but not statistically significant association with increased EBL (P = 0.097) and transfusions (P = 0.086).

Postoperative bleeding in one and fascial dehiscence in another led to reoperation in two patients (5%). Five patients (13%) had various other Clavien–Dindo grades III–IV complications, but none died. Although four patients (11%) had a biochemical pancreatic leak, only two (5%) progressed to postoperative POPF, and zero had a bile leak. Two patients (5%) developed an SSI, and one of them also had POPF, suggesting a positive association between POPF and SSI (chi-square P = 0.004). Older age was negatively associated with SSI (P = 0.048).

POPF was positively associated with DGE (chi-square P = 0.035). DGE occurred in 10 patients (26%), and most were grade B (Table 3). One patient had both POPF and DGE (grade C), one had only POPF, nine had only DGE (three grade A, five grade B, one grade C), and 27 had neither. DGE was associated with an increased hospital LOS, but POPF was not. The average LOS was 11 ± 5 days for the 10 patients with DGE, whereas it was 7 ± 3 days for the 28 without (P = 0.035). The average LOS was 12 ± 6 days for the two patients with POPF, whereas it was 8 ± 4 days for the 36 without (P = 0.165). CD III–IV had a positive trend toward increased LOS (P = 0.187). The average initial LOS for all patients with PD was 8 ± 4 days.

The readmission rate was 21% and positively associated with pancreatic leak (P = 0.005), POPF (P = 0.005), SSI (P = 0.005), and CD III–IV (P < 0.001). Three patients had both readmission and pancreatic leak, five had readmission without a leak, one had a leak without readmission, and 19 had neither. Two patients had both readmission and POPF, six had readmission without POPF, none had POPF without readmission, and 30 had neither. The SSI pattern was the same as that of POPF, even though not all patients with SSI had POPF nor vice versa. Four patients had both readmission and CD III–IV, four had readmissions without a CD III–IV, one had a CD III–IV without readmission, and 29 had neither. Interestingly, smoking was associated with a lower readmission rate than non-smoking (P < 0.001). Age, sex, chronic obstructive pulmonary disease, diabetes, cardiac disease, body mass index (BMI), and cancer status had no statistically significant effect on the readmission rate.

### Non-pancreaticoduodenectomy surgical patients

DPSs were divided between five open (including one conversion) and 11 robotic cases for a total of 16, comprising 13% of all HPB cases. This patient group had 31% men, 56% smokers, 6% with chronic obstructive pulmonary disease, 13% with cardiac disease, 38% with diabetes, and an average BMI of 29 kg/m^2^. DPSs were performed for malignancy in a total of nine cases (56%), which were segregated into three open operations for adenocarcinoma and six robotic ones for neuroendocrine tumor. Consequently, R0 resection was achieved in all nine of these patients. Except for one open approach for pancreatico-colonic fistula with pancreatic necrosis, benign indications for surgery such as mucinous cystic and branch-duct intraductal papillary mucinous neoplasms were all approached robotically. There was one conversion to open surgery, which was for visualization purposes during resection because the tumor location and patient’s body habitus prevented safe dissection on the superior border of the pancreas to expose the portal vein.

Averages for DPS were an operative time of 286 min (244 for open, 306 for robotic), EBL of 306 mL (460 for open, 236 for robotic), and intraoperative transfusion requirement of 0.2 packed red blood cell units (± 0.5 for open, ± 0.6 robotic). Differences in these outcomes based on the surgical approach were not statistically significant (Table 4). Averages of these outcomes improved over time from the first half to the latter half of our patients (median values were excluded because of an odd number of cases): operative time in minutes from 264 to 228 for open (P = 0.730) or 370 to 261 for robotic (P = 0.029), and EBL in mL from 575 to 500 for open (P = 0.862) or 330 to 170 for robotic (P = 0.191). Only improvement of the robotic operative time was statistically significant.

**Table 4:** Outcomes of non-pancreaticoduodenectomy pancreatic surgical patients

Outcome	DPS N (of 16)	PNCG N (of 7)	PNRCJ N (of 1)	DPPHR N (of 1)	TP N (of 1)
R1 resection	0 (*0%)	–	–	–	–
Reoperation	0 (0%)	0 (0%)	0 (0%)	0 (0%)	0 (0%)
Chem Leak	5 (31%)	0 (0%)	0 (0%)	0 (0%)	–
POPF	0 (0%)	0 (0%)	0 (0%)	0 (0%)	–
Bile Leak	–	–	–	0 (0%)	0 (0%)
SSI	0 (0%)	0 (0%)	0 (0%)	0 (0%)	0 (0%)
DGE	0 (0%)	0 (0%)	0 (0%)	0 (0%)	0 (0%)
CD III–IV	0 (0%)	1 (14%)	0 (0%)	0 (0%)	1 (100%)
Readmission	2 (13%)	1 (14%)	1 (100%)	1 (100%)	0 (0%)
Mortality	0 (0%)	0 (0%)	0 (0%)	0 (0%)	0 (0%)

*CD III–IV* Clavien–Dindo grades III–IV complication, *Chem Leak* Biochemical pancreatic leak, *DGE* Delayed gastric emptying, *DPPHR* Duodenum-preserving pancreatic head resection, *DPS* Distal pancreatectomy with splenectomy, *PNCG* Transgastric pancreatic necrosectomy with cyst gastrostomy, *PNRCJ* Pancreatic necrosectomy with Roux-en-Y cyst jejunostomy, *POPF* Grade B or C postoperative pancreatic fistula, *R1* Microscopic residual tumor, *SSI* Surgical site infection, *TP* Total pancreatectomy

*This rate only applies to the nine DPS cases performed for cancer

Outcomes are demonstrated in Table 4. DPS did not lead to any instances of reoperation, SSI, DGE, CD III–IV, or mortality. The open versus robotic approach outcomes are described in Table 5. Although the biochemical pancreatic leak rate was 31% (0% for open, 45% for robotic, P = 0.119), there was no case of POPF. Though biochemical pancreatic leak had a statistically significant association with readmission after PD, the difference in the readmission rate between open and robotic DPSs (0% and 18%, respectively) was not statistically significant (P = 0.308), perhaps because of the small sample size. The reasons for readmitting the two patients after DPS were as follows: vasovagal syncope during drain removal in the setting of anemia of 9.9 g/dL (on home oral anticoagulation) and pain control in the setting of dehydration (the patient had chronic analgesic and psychotropic drug use, and the immediate postoperative course was complicated by generalized ileus without pancreatic leak). The average initial hospital LOS was 6 days for both open and robotic DPSs.

**Table 5:** Outcomes of patients with distal pancreatectomy with splenectomy by approach

Outcome	Open N (of 5)	Robotic N (of 11)	P-value
R1 Resection	0 (*0%)	0 (*0%)	–
Reoperation	0 (0%)	0 (0%)	–
Chem Leak	0 (0%)	5 (45%)	0.119
POPF	0 (0%)	0 (0%)	–
Bile Leak	0 (0%)	0 (0%)	–
SSI	0 (0%)	0 (0%)	–
DGE	0 (0%)	0 (0%)	–
CD III–IV	0 (0%)	0 (0%)	–
Readmission	0 (0%)	2 (18%)	0.308
Mortality	0 (0%)	0 (0%)	–
Average OR Time	244 ± 67 min	306 ± 85 min	0.175
Average EBL	460 ± 323 mL	236 ± 183 mL	0.095
Average PRBC	0.2 ± 0.5 units	0.2 ± 0.6 units	0.953
Average LOS	6.0 ± 1.7 days	6.0 ± 1.5 days	1.000
Median LOS	5 days	6 days	–

*CD III–IV* Clavien–Dindo grades III–IV complication, *Chem Leak* biochemical pancreatic leak, *DGE* delayed gastric emptying, *EBL* estimated blood loss, *LOS* initial hospital length of stay, *OR Time* operative time, *POPF* grade B or C postoperative pancreatic fistula, *PRBC* packed red blood cells transfused intraoperatively, *R1* microscopic residual tumor, *SSI* surgical site infection

*This rate only applies to the three open and six robotic cases performed for cancer

PNCGs were performed between March 2019 through March 2020, and all seven (6% of all HPB cases in this study) were performed robotically. This patient group had 57% men, 71% smokers, 14% with chronic obstructive pulmonary disease, 29% with cardiac disease, 29% with diabetes, and an average BMI of 30 kg/m^2^. The average operative time was 190 min, average EBL was 40 mL, and there was no requirement for transfusion or reoperation. There were no cases of biochemical pancreatic leak, POPF, SSI, or DGE (Table 4). The single CD III–IV was a gastrointestinal hemorrhage in a patient who had resumed home oral anticoagulation (apixaban), leading to readmission for endoscopic control of the bleeding cyst-gastrostomy suture line. The average LOS was 3 days. Median LOS data were available for three PNCG cohorts of HVAS: 7.0 days for 20 patients [17], 14.5 days for 88 patients [18], and 13.0 days for 91 patients [18]. The LOS was lower for the CCS (3.1 ± 0.1) than for HVAS (13.1 ± 2.2; P < 0.001).

Outcomes of other open surgery pancreas patients are described in Table 4. PNRCJ was performed for a 25-cm pancreatic pseudocyst that was causing a large bowel obstruction. The patient was a male smoker with a BMI of 27 kg/m^2^. The operative time was 178 min with an EBL of 50 mL and no requirement for transfusion or reoperation. There were no major postoperative complications. The initial hospital LOS was 6 days. He was readmitted because of dehydration.

Duodenum-preserving pancreatic head resection was performed in one patient for chronic calcific pancreatitis. The patient was a male smoker with chronic obstructive pulmonary disease, diabetes, and a BMI of 25 kg/m^2^. The operative time was 362 min with an EBL of 300 mL and no requirement for transfusion or reoperation. There were no major postoperative complications. The LOS was 6 days, and he was readmitted for pain control.

Total pancreatectomy was performed in one patient for two branch-duct intraductal papillary mucinous neoplasms with high-grade dysplasia in the head and body of the pancreas. The patient was a male smoker with a BMI of 30 kg/m^2^. The operative time was 532 min with an EBL of 400 mL and no requirement for transfusion or reoperation. He did have a CD III–IV on postoperative day 3, which was an embolic stroke treated medically. This lengthened his LOS to 26 days, but he did not require readmission.

## Discussion

### All pancreatic surgical patients

The principal statistical analyses were comparisons of mixed pancreatic surgical patient outcomes between seven literature-based HVAS and the CCS, and they showed several key findings. First, the CCS and HVAS PD and DP proportions were similar, indicating that the pancreatic surgical case mix was comparable. Second, using comparative analysis of the ASA ≥ 3 proportion, the evidence demonstrated that the CCS patients had more severe medical comorbidities at the time of pancreatic surgery than the patients of HVAS. Third, compared to the patients of HVAS, the CCS patients had comparable proportions for 90-day mortality, DGE, and readmission. Fourth, the CCS patients had lower proportions for POPF, reoperation, SSI, and CD III–IV than the patients of HVAS. These findings, despite the higher preoperative comorbidity, indicate that the patient outcomes of the CCS were comparable to those of high-volume academic centers, which confirms our hypothesis. We believe that the development of an effective pancreatic surgical program in a community hospital includes three key elements: (1) the availability of a motivated and well-trained surgeon; (2) selection of a community hospital that has demonstrated operational finesse (e.g., an accredited surgical training program and verified trauma center); and (3) the development of complementary quality enhancement programs. The evidence from the current study comparisons indicates that a quality pancreatic surgical program can be developed in a community hospital that has historically demonstrated a commitment for establishing services of excellence.

### Patients with pancreaticoduodenectomy

The most common pancreatic operation was PD, for which many outcomes were improved compared to those reported by high-volume academic centers. The R1 resection rate was 18% vs. the reported 24–30% [19, 20], reoperation rate was 5 vs. 8% [21], bile leak rate was 0 vs. 4% [21], SSI rate was 5 vs. 11% [22], and CD III–IV rate was 13 vs. 21–30% [1, 21], respectively. The DGE rate was equivalent at 26% compared to 9–31% [19, 21, 22]. Despite the associations between DGE and a longer initial hospital LOS, there was a low LOS average: 8 (median 6) days vs. the literature-reported average of 8–12 (median 8–11) days [2, 19–23].

The postoperative POPF rate of PD in the CCS was also lower at 5% vs. the 10–19% reported in the literature [1, 2, 19, 22], although our asymptomatic biochemical pancreatic leak rate was higher at 11% vs. 6% [21]. Consistent with the literature and the current analysis, we found a positive association between biochemical leak and readmission (P = 0.005). The CCS also demonstrated a higher readmission rate of 21% vs. 10–19% in HVAS [20, 23]. However, the most important outcome and one that is commonly used to base the reason for centralization of HPB care is mortality, for which we had a rate of 0% compared to the 1–7% reported by high-volume academic centers [2, 19–24]. Long-term analysis at the 5-, 7-, and 10-year marks will hopefully continue to reflect those rates nationally.

When comparing the average operative metrics for patients with PD in the current studies’ second and third years to published values, we found that the operative time was 403 min vs. 253–335 min, and EBL was 501 mL vs. 350–780 mL, respectively [2, 19, 23]. Reports of transfusion requirements were harder to find. The CCS average was 1.0 ± 1.6 packed red blood cell units given intraoperatively (reduced to 0.6 units by the latter half of our study). The median, however, was 0 units given intraoperatively vs. a median of 3 units given within 72 h of surgery according to national data from 2005 to 2008 reported by Ball et al. [25]. Improvements in operative time and EBL were statistically significant, and may reflect the learning curve of both the surgeon and the specific operating room staff (including a cardiac-specific anesthesiologist) designated from the beginning to assist with all PD surgeries.

Consistency with intraoperative and postoperative care may be the reason for achieving success with a shorter LOS, low rates of complications, and 0% mortality. Patients receiving any type of pancreatic surgery (not just PD) were admitted to either the surgical intensive care unit or a designated step-down unit specified prior to the start of this program. All patients with PD were admitted to the surgical intensive care unit postoperatively for a minimum of 24 h. Nurses in these units were familiarized with care involving the pancreas, and followed a tailored protocol developed by our HPB surgeon based on the pancreatic enhanced recovery after surgery recommendations [3].

### Non-pancreaticoduodenectomy surgical patients

Patients receiving DPS usually followed the protocol in a more expedited fashion, as reflected in the average LOS of 6 days. There were only five open DPS cases in the CCS with a 0% morbidity and mortality rate for these patients thus far. A high-volume academic hospital in our neighboring city reported 21 open DPS cases with rates of 11% for R1 resection, 14% for reoperation, 14% for POPF, 24% for SSI, 0% for DGE, 29% for CD III–IV, 29% for readmission, and 5% for 60-day mortality [12]. The median EBL of 300 mL with a median LOS of 5 days for our open cases was also equivalent to their data [12]. There were no comparisons available for intraoperative transfusion [12].

The patients with robotic DPS of the CCS had few complications, but rates were comparable to those published by two academic centers with expertise in robotic DP (with selective splenic preservation). Of the 11 cases in the CCS, 45% had biochemical pancreatic leak without progression to POPF, whereas high-volume centers had a 28% leak rate with a separate 12–19% POPF rate [26, 27]. One patient’s readmission seemed unrelated (vasovagal syncope and anemia of 9.9 g/dL on anticoagulation). The robotic DPS group had no R1 resections (adenocarcinomas were not treated robotically) or other complications aside from asymptomatic leaks and readmission at a rate of 18%, which is equivalent to the 20–25% readmission rate at high-volume centers [26, 27]. Previous studies reported rates of 5–22% for R1 resection, 2% for reoperation, 72% for CD III–IV, and 0–5% for 90-day mortality [26, 27]. Our institution is optimistic in closing the gap within other metrics as experience increases: our average operative time of 306 min vs. their 210–252 min, average EBL of 236 mL vs. 150–406 mL, and median LOS of 6 days vs. 5 days [26, 27].

Finally, the CCS showed that seven robotic PNCG surgeries were performed safely. One patient was readmitted for endoscopic control of the bleeding cyst-gastrostomy suture line. The small patient volume resulted in a 14% rate of CD III–IV with a readmission. However, there were no mortalities. For comparison, a high-volume academic center with experience in 14 robotic and six laparoscopic PNCG cases reported rates of 5% for bleeding, 15% for reoperation for residual necrosis, 10% for infection, and 0% for mortality [17]. The average and median LOS of the CCS were significantly less than those found in three other cohorts of HVAS [17, 18]. Other outcomes can be put into perspective by considering the recently published data from 91 patients at a large high-volume center receiving a minimally invasive step-up approach to treat necrotizing pancreatitis: 19%, re-intervention rate for bleeding; 66%, rate of POPF; 63%, rate of readmission; and 2%, rate of 90-day mortality [18].

One limitation of the present study is the small sample size of patients from a level one trauma center with a community teaching residency program that is still in its infancy. Additionally, there was a selection bias between the robotic and open approaches, which likely factored into our outcomes. However, the overall patient population and case complexity of the CCS remained diverse and comparable to those of high-volume centers. As the patient volume continues to increase, long-term analysis will be needed at the 5, 7, and 10-year marks to ensure that the outcomes are still equivalent.

## Conclusions

Particularly in cases of pancreatic cancer, the care is complex and involves numerous perioperative clinic appointments as well as treatment visits for chemotherapy and radiation often multiple times per week. Access to care and proximity to home with social support systems are important for patients with a stressful diagnosis. Local patients who previously received major pancreatic surgery at one of our neighboring high-volume centers also occasionally visit us for postoperative concerns upon learning about the institution’s program. This further emphasizes the utility of establishing a local HPB center for continuity of care. A well-trained and cohesive team of multidisciplinary physicians and support staff can expand access to high-quality care for broader patient communities.

Furthermore, an enhanced recovery after surgery protocol tailored to pancreatic surgeries achieved a median index LOS that was 25–50% shorter than that of academic centers after PD. Rates of R1 resection, reoperation, mortality, POPF, bile leak, and SSI after PD were also improved compared to those of academic centers. Despite the learning curve, robotic pancreatic surgeries were safely incorporated into our new program. Thanks to appropriate hospital infrastructure with a trained HPB surgeon, protocols to guide care, and a good multidisciplinary team, our newly established medium-volume community program was able to demonstrate outcomes equivalent to those of academic high-volume centers in only 3 years.

## Supplementary Information

**Additional file 1. **Proportions of patients with pancreaticoduodenectomy and distal pancreatectomy in high-volume academic centers. Table showing the proportions of patients with pancreaticoduodenectomy and distal pancreatectomy in high-volume academic centers.

**Additional file 2. **Proportions of patients with an American Society of Anesthesiologists physical status ≥3 in high-volume academic centers. Table showing the proportions of patients with an American Society of Anesthesiologists status ≥3 in high-volume academic centers.

**Additional file 3.** Proportions of patients with 90-day mortality in high-volume academic centers. Table showing the proportions of patients with 90-day mortality in high-volume academic centers.

**Additional file 4. **Proportions of patients with postoperative pancreatic fistula in high-volume academic centers. Table showing the proportions of patients with postoperative pancreatic fistula in high-volume academic centers.

**Additional file 5. **Proportions of patients with reoperation in high-volume academic centers. Table showing the proportions of patients with reoperation in high-volume academic centers.

**Additional file 6. **Proportions of patients with surgical site infection in high-volume academic centers. Table showing the proportions of patients with surgical site infection in high-volume academic centers.

**Additional file 7. **Proportions of patients with postoperative delayed gastric emptying in high-volume academic centers. Table showing the proportions of patients with postoperative delayed gastric emptying in high-volume academic centers.

**Additional file 8. **Proportions of patients with postoperative Clavien–Dindo grades III–IV complication in high-volume academic centers. Table showing the proportions of patients with postoperative Clavien–Dindo grades III–IV complication in high-volume academic centers.

**Additional file 9. **Proportions of patients with readmission in high-volume academic centers. Table showing the proportions of patients with readmission in high-volume academic centers.

## Data Availability

All relevant data are within the manuscript and its Additional files. The datasets generated and/or analyzed during the current study are not publicly available as they consist of confidential patient data; however, data will be made available from the corresponding author on reasonable request.

## Abbreviations

HPB: Hepato-pancreato-biliary
SEYH: St. Elizabeth Youngstown Hospital
DPS: Distal pancreatectomy with splenectomy
PNCG: Pancreatic necrosectomy with cyst gastrostomy
PD: Pancreaticoduodenectomy
EBL: Estimated blood loss
POPF: Postoperative pancreatic fistula
SSI: Surgical site infection
DGE: Delayed gastric emptying
CD III–IV: Clavien–Dindo grades III–IV complication
LOS: Length of stay
ASA: American Society of Anesthesiologists physical status
HVAS: High-volume academic center studies
CCS: Current community study
DP: Distal pancreatectomy
PNRCJ: Pancreatic necrosectomy with Roux-en-Y cyst jejunostomy
BMI: Body mass index
R1: Microscopic residual tumor
