# Supplementary material for: Establishing an open and robotic pancreatic surgery program in a level 1 trauma center community teaching hospital and comparing its outcomes to high-volume academic center outcomes: a retrospective review
Source: BMC Surg. 2022 Dec 6;22:414. doi: 10.1186/s12893-022-01867-7 (PMC9724418; doi:10.1186/s12893-022-01867-7)
Supplement: Supplementary file 1 — Additional file 1. Proportions of patients with pancreaticoduodenectomy and distal pancreatectomy in high-volume academic centers. Table showing the proportions of patients with pancreaticoduodenectomy and distal pancreatectomy in high-volume academic centers. [file 12893_2022_1867_MOESM1_ESM.docx]

**Additional file 1. Proportions of PD and DP patients in high-volume academic centers.**

| **Study** | **PD** | **Total** | **%** | **DP** | **Total** | **%** |
| --- | --- | --- | --- | --- | --- | --- |
| Gabel, 2020 [10] | 115 | 173 | 66.5 | 56 | 173 | 32.4 |
| Hanna-Sawires, 2019 [11] | 160 | 240 | 66.7 | 57 | 240 | 23.8 |
| Hardacre, 2015 [12] | 15 | 28 | 53.6 | 11 | 28 | 39.3 |
| Nicholas, 2021 [14] | 182 | 273 | 66.7 | 91 | 273 | 33.3 |
| Salvia, 2021 [15] | 827 | 1230 | 67.2 | 403 | 1230 | 32.8 |
| Schlottmann, 2015 [16] | 48 | 73 | 65.8 | 25 | 73 | 34.2 |

Abbreviations: PD, pancreaticoduodenectomy; DP, distal pancreatectomy

Test for PD proportion heterogeneity: P = 0.8001

Total PD proportion: 66.7%

Test for DP proportion heterogeneity: P = 0.0906

Total DP proportion: 31.9%
