# Supplementary material for: Establishing an open and robotic pancreatic surgery program in a level 1 trauma center community teaching hospital and comparing its outcomes to high-volume academic center outcomes: a retrospective review
Source: BMC Surg. 2022 Dec 6;22:414. doi: 10.1186/s12893-022-01867-7 (PMC9724418; doi:10.1186/s12893-022-01867-7)
Supplement: Supplementary file 2 — Additional file 2. Proportions of patients with an American Society of Anesthesiologists physical status ≥3 in high-volume academic centers. Table showing the proportions of patients with an American Society of Anesthesiologists status ≥3 in high-volume academic centers. [file 12893_2022_1867_MOESM2_ESM.docx]

**Additional file 2. Proportions of patients with an American Society of Anesthesiologists physical status ≥3 in high-volume academic centers.**

| **Study** | **ASA ≥3** | **Total** | **ASA ≥3** |
| --- | --- | --- | --- |
| Hanna-Sawires, 2019 [11] | 46 | 240 | 19.2% |
| Hardacre, 2015 [12] | 21 | 28 | 75.0% |
| Salvia, 2021 [15] | 241 | 1230 | 19.6% |
| Schlottmann, 2015 [16] | 12 | 73 | 16.4% |

*Abbreviation:* ASA, American Society of Anesthesiologists physical status

Test for proportion heterogeneity: P < 0.0001

Total proportion: 28.1%
