# Supplementary material for: Establishing an open and robotic pancreatic surgery program in a level 1 trauma center community teaching hospital and comparing its outcomes to high-volume academic center outcomes: a retrospective review
Source: BMC Surg. 2022 Dec 6;22:414. doi: 10.1186/s12893-022-01867-7 (PMC9724418; doi:10.1186/s12893-022-01867-7)
Supplement: Supplementary file 3 — Additional file 3. Proportions of patients with 90-day mortality in high-volume academic centers. Table showing the proportions of patients with 90-day mortality in high-volume academic centers. [file 12893_2022_1867_MOESM3_ESM.docx]

**Additional file 3. Proportions of patients with 90-day mortality in high-volume academic centers.**

| **Study** | **Died** | **Total** | **%** |
| --- | --- | --- | --- |
| Gabel, 2020 [10] | 1 | 173 | 0.6% |
| Salvia, 2021 [15] | 31 | 1230 | 2.5% |

Test for proportion heterogeneity: P = 0.0884

Total proportion: 2.3%
