# Supplementary material for: Establishing an open and robotic pancreatic surgery program in a level 1 trauma center community teaching hospital and comparing its outcomes to high-volume academic center outcomes: a retrospective review
Source: BMC Surg. 2022 Dec 6;22:414. doi: 10.1186/s12893-022-01867-7 (PMC9724418; doi:10.1186/s12893-022-01867-7)
Supplement: Supplementary file 4 — Additional file 4. Proportions of patients with postoperative pancreatic fistula in high-volume academic centers. Table showing the proportions of patients with postoperative pancreatic fistula in high-volume academic centers. [file 12893_2022_1867_MOESM4_ESM.docx]

**Additional file 4. Proportions of patients with postoperative pancreatic fistula in high-volume academic centers.**

| **Study** | **Fistula** | **Total** | **%** |
| --- | --- | --- | --- |
| Gabel, 2020 [10] | 36 | 173 | 20.8% |
| Hanna-Sawires, 2019 [11] | 38 | 240 | 15.8% |
| Hardacre, 2015 [12] | 2 | 28 | 7.1% |
| Krautz, 2019 [13] | 75 | 641 | 11.7% |
| Nicholas, 2021 [14] | 21 | 273 | 7.7% |
| Salvia, 2021 [15] | 311 | 1230 | 25.3% |
| Schlottmann, 2015 [16] | 34 | 73 | 46.6% |

Test for proportion heterogeneity: P < 0.0001

Total proportion: 18.4%
