# Supplementary material for: Establishing an open and robotic pancreatic surgery program in a level 1 trauma center community teaching hospital and comparing its outcomes to high-volume academic center outcomes: a retrospective review
Source: BMC Surg. 2022 Dec 6;22:414. doi: 10.1186/s12893-022-01867-7 (PMC9724418; doi:10.1186/s12893-022-01867-7)
Supplement: Supplementary file 5 — Additional file 5. Proportions of patients with reoperation in high-volume academic centers. Table showing the proportions of patients with reoperation in high-volume academic centers. [file 12893_2022_1867_MOESM5_ESM.docx]

**Additional file 5. Proportions of patients with reoperation in high-volume academic centers.**

| **Study** | **Reoperation** | **Total** | **%** |
| --- | --- | --- | --- |
| Hanna-Sawires, 2019 [11] | 29 | 240 | 12.1% |
| Hardacre, 2015 [12] | 1 | 28 | 3.6% |
| Salvia, 2021 [15] | 100 | 1230 | 8.1% |

Test for proportion heterogeneity: P = 0.1152

Total proportion: 8.7%
