# Supplementary material for: Establishing an open and robotic pancreatic surgery program in a level 1 trauma center community teaching hospital and comparing its outcomes to high-volume academic center outcomes: a retrospective review
Source: BMC Surg. 2022 Dec 6;22:414. doi: 10.1186/s12893-022-01867-7 (PMC9724418; doi:10.1186/s12893-022-01867-7)
Supplement: Supplementary file 6 — Additional file 6. Proportions of patients with surgical site infection in high-volume academic centers. Table showing the proportions of patients with surgical site infection in high-volume academic centers. [file 12893_2022_1867_MOESM6_ESM.docx]

**Additional file 6. Proportions of patients with surgical site infection in high-volume academic centers.**

| **Study** | **SSI** | **Total** | **%** |
| --- | --- | --- | --- |
| Gabel, 2020 [10] | 27 | 173 | 15.6% |
| Hardacre, 2015 [12] | 3 | 28 | 10.7% |
| Salvia, 2021 [15] | 434 | 1230 | 35.3% |

*Abbreviation:* SSI, surgical site infection

Test for proportion heterogeneity: P < 0.0001

Total proportion: 21.1%
