# Supplementary material for: Establishing an open and robotic pancreatic surgery program in a level 1 trauma center community teaching hospital and comparing its outcomes to high-volume academic center outcomes: a retrospective review
Source: BMC Surg. 2022 Dec 6;22:414. doi: 10.1186/s12893-022-01867-7 (PMC9724418; doi:10.1186/s12893-022-01867-7)
Supplement: Supplementary file 7 — Additional file 7. Proportions of patients with postoperative delayed gastric emptying in high-volume academic centers. Table showing the proportions of patients with postoperative delayed gastric emptying in high-volume academic centers. [file 12893_2022_1867_MOESM7_ESM.docx]

**Additional file 7. Proportions of patients with postoperative delayed gastric emptying in high-volume academic centers.**

| **Study** | **DGE** | **Total** | **%** |
| --- | --- | --- | --- |
| Gabel, 2020 [10] | 17 | 173 | 9.8% |
| Hanna-Sawires, 2019 [11] | 37 | 240 | 15.4% |
| Hardacre, 2015 [12] | 0 | 28 | 0% |
| Krautz, 2019 [13] | 61 | 641 | 9.5% |
| Salvia, 2021 | 140 | 1230 | 11.4% |
| Schlottmann, 2015 | 7 | 73 | 9.6% |

*Abbreviation:* DGE, delayed gastric emptying

Test for proportion heterogeneity: P = 0.0219

Total proportion: 10.6%
