# Supplementary material for: Establishing an open and robotic pancreatic surgery program in a level 1 trauma center community teaching hospital and comparing its outcomes to high-volume academic center outcomes: a retrospective review
Source: BMC Surg. 2022 Dec 6;22:414. doi: 10.1186/s12893-022-01867-7 (PMC9724418; doi:10.1186/s12893-022-01867-7)
Supplement: Supplementary file 8 — Additional file 8. Proportions of patients with postoperative Clavien–Dindo grades III–IV complication in high-volume academic centers. Table showing the proportions of patients with postoperative Clavien–Dindo grades III–IV complication in high-volume academic centers. [file 12893_2022_1867_MOESM8_ESM.docx]

**Additional file 8. Proportions of patients with postoperative Clavien-Dindo grades III–IV complication in high-volume academic centers.**

| **Study** | **CD III–IV** | **Total** | **%** |
| --- | --- | --- | --- |
| Hanna-Sawires, 2019 [11] | 75 | 240 | 31.3% |
| Hardacre, 2015 [12] | 2 | 28 | 7.1% |
| Nicholas, 2021 [14] | 59 | 273 | 21.6% |

*Abbreviation:* CD III–IV, Clavien-Dindo grades III–IV complication

Test for proportion heterogeneity: P = 0.0020

Total proportion: 21.8%
