# Supplementary material for: Establishing an open and robotic pancreatic surgery program in a level 1 trauma center community teaching hospital and comparing its outcomes to high-volume academic center outcomes: a retrospective review
Source: BMC Surg. 2022 Dec 6;22:414. doi: 10.1186/s12893-022-01867-7 (PMC9724418; doi:10.1186/s12893-022-01867-7)
Supplement: Supplementary file 9 — Additional file 9. Proportions of patients with readmission in high-volume academic centers. Table showing the proportions of patients with readmission in high-volume academic centers. [file 12893_2022_1867_MOESM9_ESM.docx]

**Additional file 9. Proportions of patients with readmission in high-volume academic centers.**

| **Study** | **ReAdm** | **Total** | **%** |
| --- | --- | --- | --- |
| Gabel, 2020 [10] | 31 | 173 | 17.9% |
| Hardacre, 2015 [12] | 3 | 28 | 10.7% |
| Nicholas, 2021 [14] | 53 | 273 | 19.4% |

*Abbreviation:* ReAdm, readmission

Test for proportion heterogeneity: P = 0.5604

Total proportion: 18.5%
